# Differentiation of acute non-ST elevation myocardial infarction and acute infarct-like myocarditis by visual pattern analysis: a head-to-head comparison of different cardiac MR techniques

**DOI:** 10.1007/s00330-023-09905-5

**Published:** 2023-07-12

**Authors:** Charlotte Jahnke, Martin Sinn, Amra Hot, Ersin Cavus, Jennifer Erley, Jan Schneider, Celeste Chevalier, Sebastian Bohnen, Ulf Radunski, Mathias Meyer, Gunnar Lund, Gerhard Adam, Paulus Kirchhof, Stefan Blankenberg, Kai Muellerleile, Enver Tahir

**Affiliations:** 1 Department of Cardiology, University Heart and Vascular Center Hamburg, University Medical Center Hamburg-Eppendorf, Martinistrasse 52, 20246 Hamburg, Germany; 2 Department of Diagnostic and Interventional Radiology and Nuclear Medicine, University Medical Center Hamburg-Eppendorf, Hamburg, Germany; 3 Department of Medical Biometry and Epidemiology, University Medical Center Hamburg-Eppendorf, Hamburg, Germany; 4 Department of Cardiology, Regio Clinics, Pinneberg, Germany; 5 German Center for Cardiovascular Research (DZHK), Partner Site Hamburg/Kiel/Lübeck, Hamburg, Germany

**Keywords:** Myocardial infarction, Myocarditis, Magnetic resonance imaging, Cardiac imaging techniques

## Abstract

**Objectives:**

Parametric cardiac magnetic resonance (CMR) techniques have improved the diagnosis of pathologies. However, the primary tool for differentiating non-ST elevation myocardial infarction (NSTEMI) from myocarditis is still a visual assessment of conventional signal-intensity-based images. This study aimed at analyzing the ability of parametric compared to conventional techniques to visually differentiate ischemic from non-ischemic myocardial injury patterns.

**Methods:**

Twenty NSTEMI patients, twenty infarct-like myocarditis patients, and twenty controls were examined using cine, T2-weighted CMR (T2w) and late gadolinium enhancement (LGE) imaging and T1/T2 mapping on a 1.5 T scanner. CMR images were presented in random order to two experienced fully blinded observers, who had to assign them to three categories by a visual analysis: NSTEMI, myocarditis, or healthy.

**Results:**

The conventional approach (cine, T2w and LGE combined) had the best diagnostic accuracy with 92% (95%CI: 81–97) for NSTEMI and 86% (95%CI: 71–94) for myocarditis. The diagnostic accuracies using T1 maps were 88% (95%CI: 74–95) and 80% (95%CI: 62–91), 84% (95%CI: 67–93) and 74% (95%CI: 54–87) for LGE, and 83% (95%CI: 66–92) and 73% (95%CI: 53–87) for T2w. The accuracies for cine (72% (95%CI: 52–86) and 60% (95%CI: 38–78)) and T2 maps (62% (95%CI: 40–79) and 47% (95%CI: 28–68)) were significantly lower compared to the conventional approach (*p* < 0.001 and *p* < 0.0001).

**Conclusions:**

The conventional approach provided a reliable visual discrimination between NSTEMI, myocarditis, and controls. The diagnostic accuracy of a visual pattern analysis of T1 maps was not significantly inferior, whereas the diagnostic accuracy of T2 maps was not sufficient in this context.

**Clinical relevance statement:**

The ability of parametric compared to conventional CMR techniques to visually differentiate ischemic from non-ischemic myocardial injury patterns can avoid potentially unnecessary invasive coronary angiography and help to shorten CMR protocols and to reduce the need of gadolinium contrast agents.

**Key Points:**

**•** *A visual differentiation of ischemic from non-ischemic patterns of myocardial injury is reliably achieved by a combination of conventional CMR techniques (cine, T2-weighted and LGE imaging).*

**•** *There is no significant difference in accuracies between visual pattern analysis on native T1 maps without providing quantitative values and a conventional combined approach for differentiating non-ST elevation myocardial infarction, infarct-like myocarditis, and controls.*

**•** *T2 maps do not provide a sufficient diagnostic accuracy for visual pattern analysis for differentiating non-ST elevation myocardial infarction, infarct-like myocarditis, and controls.*

## Introduction

The clinical presentation of acute myocarditis is highly variable [1]. Clinical discrimination between acute “infarct-like” myocarditis and acute non-ST elevation myocardial infarction (NSTEMI) can be challenging as initial presentation and clinical findings may be similar and not specific enough to distinguish between both diseases without coronary angiography. Especially in young patients with a low coronary risk profile, an early cardiac magnetic resonance (CMR) imaging approach is desirable to avoid potentially unnecessary invasive coronary angiography [2].

CMR is the reference technique for non-invasive myocardial tissue characterization [3]. Conventional signal-intensity-based sequences provide a visual qualitative and semi-quantitative assessment of both ischemic and non-ischemic myocardial injury [4, 5]. So, until recently, to diagnose myocardial infarction, late gadolinium enhancement (LGE) imaging and T2-weighted (T2w) CMR were used to visualize scar and/or acute ischemic necrosis [2, 6]. Likewise, the diagnosis of myocarditis was based on the Lake Louise criteria (LLC), including conventional T2w imaging, early gadolinium enhancement (EGE), and LGE [7]. Of note, especially the assessment of pattern and localization of myocardial damage on LGE images allows a differentiation between ischemic and non-ischemic myocardial injury which is the key feature of CMR in this context [2, 4].

However, the detection of diffuse myocardial pathologies by conventional CMR techniques is limited [8, 9]. Recently, parametric T1 and T2 mapping approaches overcame this limitation by directly quantifying myocardial T1 and T2 relaxation times and have been progressively integrated into routine CMR imaging [10, 11]. Hence, in 2018 the LLC were updated by including parametric mapping techniques [12]. Further improvements of these techniques led to deeper characterization and complimentary information, e.g., the possibility to identify additional areas of myocardial damage beyond LGE and T2w CMR [13].

For that reason, many centers completely replaced T2w imaging by T1 and/or T2 mapping, which carries the risk for missing focal myocardial tissue alterations. Therefore, aim of our study was to assess whether parametric CMR techniques, with all their potential for quantitative tissue characterization, are also capable to differentiate ischemic from non-ischemic patterns of myocardial injury similar to conventional signal-intensity-based LGE and T2w imaging by a simple visual pattern analysis, i.e., without providing quantitative values. This could help to shorten CMR protocols by omitting conventional T2w imaging and ultimately even to avoid the need of gadolinium contrast agents by omitting LGE imaging.

## Materials and methods

### Patients and controls

The population of this retrospective study included a composed collective of 60 subjects: 20 patients each with acute NSTEMI and acute “infarct-like” myocarditis who both clinically presented with chest pain and a control group of 20 healthy subjects, respectively. The subjects were initially recruited between April 2011 and January 2016 for recently published studies, approved by the local ethics committee and informed written consent by the participants [14, 15].

The NSTEMI group (median age: 55 (53–65) years, 85% male) (Table 1) consisted of patients with reperfused NSTEMI, which was defined by using the established diagnostic criteria (increase of Troponin values above the 99th percentile, clinical symptoms of myocardial ischemia and/or new ischemic changes in electrocardiogram (ECG)) [16]. The myocarditis group (median age: 34 (31–44) years, 85% male) (Table 1) was defined clinically and included patients with acute chest pain in the absence of coronary artery disease, with a history of recent gastrointestinal and/or airway infection and dynamically elevated troponin T and/or N-terminal pro-B-type natriuretic peptide levels [14, 17]. Exclusion criteria in the myocarditis group were patients who fulfilled the clinical criteria of myocarditis but presented with a phenotype of a new-onset heart failure with reduced left ventricular (LV) ejection fraction (EF) (“cardiomyopathy-like” myocarditis) [17]. Moreover, all patients with concomitant coronary artery disease, severe LV hypertrophy, takotsubo cardiomyopathy, and hereditary dilated cardiomyopathy were excluded [14].

**Table 1:** Major clinical characteristics of patients and controls

	Controls (*n* = 20)	NSTEMI (*n* = 20)	Myocarditis (*n* = 20)	*p value for NSTEMI vs. myocarditis*
Clinical parameters				
Age, years	40 (35–46)	55 (53–65)^‡^	34 (31–44)	** < *0.0001***
Male, %	14 (70)	17 (85)	17 (85)	*1.00*
Troponin T, pg/mL	3 (3–5)	785 (394–1129)^‡^	432 (289–637)^‡^	*0.51*
CK max, U/l	145 (116–200)	294 (255–581)*	223 (202–410)*	*0.53*
Triglycerides mg/dL		109 (88–223)	148 (100–171)	*0.90*
Cholesterol mg/dL		181 (139–213)	168 (147–212)	*0.80*
LDL-Cholesterol mg/dL		96 (77–115)	111 (80–124)	*0.44*
HDL-Cholesterol mg/dL		41(35–47)	40 (27–50)	*0.72*
CMR interval, days		8 (6–10)	15 (2–27)	*0.53*
CMR parameters				
LVEF, %	64 (62–67)	59 (54–64)	55 (49–60)^†^	*0.32*
LVEDVi, mL/m^2^	146 (137–164)	145 (133–168)	163 (142–191)	*0.24*
LVESVi, mL/m^2^	48 (52–73)	59 (52–73)	65 (55–105)	*0.29*
LVSVi, mL/m^2^	93 (86–106)	94 (81–96)	93 (79–95)	*0.90*

Values are median (interquartile range) or *n* (%)

^*^p < 0.05, ^†^*p* < 0.01 or ^‡^*p* < 0.0001 for NSTEMI or myocarditis patients vs. controls

*CK*, creatine kinase; *CMR*, cardiac magnetic resonance; *LVEDVi*, left ventricular end-diastolic volume index; *LVEF*, left ventricular ejection fraction; *LVESVi*, left ventricular end-systolic volume index; *LVSVi*, left ventricular stroke volume index

### CMR protocol

CMR was performed on a 1.5 T scanner equipped with a 5-channel cardiac phased array receiver coil (Achieva, Philips Medical Systems) at 8 (6–10 interquartile range (IQR)) days (NSTEMI) and 15 (2–27 IQR) days (myocarditis) following onset of symptoms. The CMR protocol included standard steady-state free-precession cine CMR in short axis with retrospective ECG triggering. An edema-sensitive black-blood T2w sequence was performed on end-diastolic LV short-axis using a fat-suppressed (STIR) triple inversion-recovery turbo-spin-echo sequence with full LV coverage. For T2 mapping, a gradient- (echo planar imaging) and spin-echo multi-echo sequence (GraSE) in three end-diastolic short-axis slices (apical, mid, and basal) was used [15]. Corresponding three native T1 mapping slices were acquired using a 3(3)5 Modified Look-Locker Inversion Recovery (MOLLI) sequence [9]. Ten minutes after a bolus injection of 0.075 mmol/kg gadobenate dimeglumine at a rate of 2.5 mL/s end-diastolic LGE images were acquired using end-diastolic phase-sensitive inversion recovery (PSIR) sequences in short-axis orientation covering the entire heart and in two-, three-, and four-chamber views. Native T1 and T2 maps were generated using a dedicated plug-in written for the OsiriX software (Pixmeo) [9]. Typical pixel sizes for the different sequences were as follows: Cine CMR: 1.4 × 1.4 × 10 mm^3^; T2-STIR CMR: 1.45 × 1.45 × 10 mm^3^; LGE CMR: 0.97 × 0.97 × 8 mm^3^; T1 mapping: 1.17 × 1.17 × 10 mm^3^; and T2 mapping: 1.05 × 1.05 × 10 mm^3^, respectively.

### CMR data analyses

**Fig. 1:**
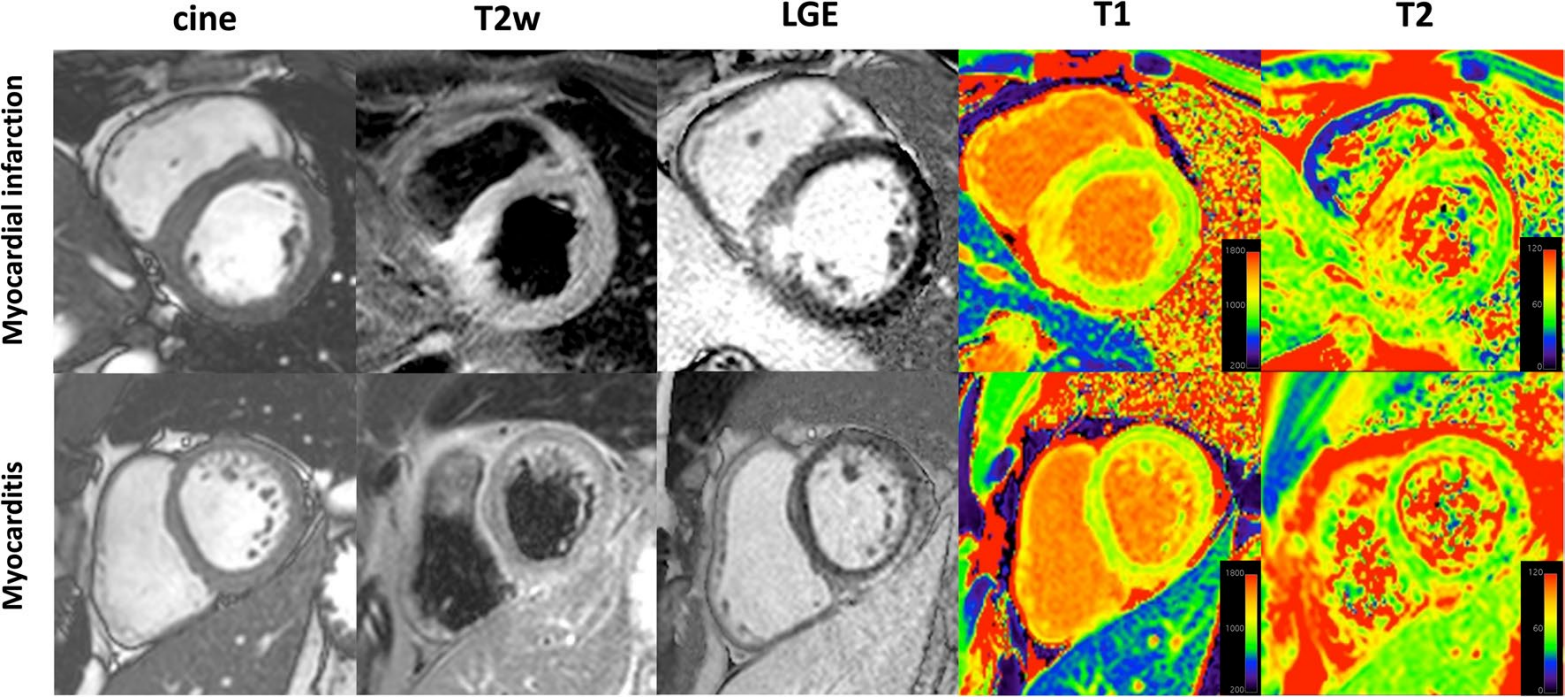
Example of typical CMR findings in a patient with NSTEMI (upper line) compared to a patient with “infarct-like” myocarditis (lower line) from the study population. T2w CMR and LGE imaging show an area with myocardial edema and necrosis (subendocardial and inferoseptal for myocardial infarction, subepicardial and inferior for myocarditis). T1 and T2 maps show corresponding increased T1 and T2 mapping times, which are presented as color changes in the respective area. Different mapping times are depicted based on the color scaling on the right side of each map (the ranges with very high mapping values have red colors while very decreased values are shown in purple)

Completely anonymized CMR images of patients with NSTEMI, myocarditis, and controls were merged in a random order in five different groups each representing a single CMR technique (cine, T2w, LGE, T1 and T2) (see Fig. 1) and in a combination of the non-parametric techniques cine, T2w and LGE as a sixth group (“conventional approach”). Two observers blinded to all CMR data with 9 years (E.T.) and > 15 years (K.M.) of experience in CMR had to assign the images to one of the three categories: myocardial infarction, myocarditis, or healthy, based on visual pattern analysis. Cine CMR was presented as cine loops of 25 LV short-axis images per slice. T2w sequences were shown as three corresponding and representative end-diastolic LV sections in short-axis orientation. LGE images were presented as three corresponding and representative LV sections in short-axis orientation as well as two-, three-, and four-chamber views. T1 and T2 maps were presented to the observers as color-coded maps for visual analysis of three short-axis slices, namely each color gradation coded for the specific and absolute relaxation time of a pixel. Figure 1 demonstrates typical CMR findings in two patients with myocardial infarction and myocarditis.

### Statistical analyses

Statistical analyses were performed using GraphPadPrism version 9.1 for Macintosh (GraphPad Software) and R Version 4.0.5 (2021–03-31) (R Foundation for Statistical Computing). Continuous data are presented as median with IQR and categorical data as absolute numbers and percentage. Differences in baseline variables between the diagnostic groups were analyzed by using the non-parametric Mann–Whitney test. Due to the available cluster data structure (*n* = 60 subjects, three conditions, six CMR image groups, two observers), models with crossed random effects were calculated with CMR as fixed effect and subject ID and observers as random effect in an explorative manner. The aim was to estimate the sensitivity and specificity of the target condition, i.e., the probability of correctly identifying diseased and non-diseased individuals. Thereby, these models were based on the patient population with and without the corresponding target condition. The estimation of the observers (true positive and true negative) was set as the dependent variable. Confidence intervals were calculated using the modified Wald method. Furthermore, the aim was to determine the probability of correctly detecting the respective condition among all subjects, i.e., the overall accuracy to compare the different CMR techniques. The same model as described above was used. The population consisted of all individuals regardless of the target condition. As a result, adjusted odds ratios with their corresponding confidence intervals are reported. These indicate the likelihood of correctly detecting the target condition with the respective CMR technique compared to the conventional approach. In this model, adjustment for multiplicity was performed by using the Bonferroni correction method. Statistical significance was set to *p* < 0.05.

## Results

Major characteristics of the study population are provided in Table 1. Patients with NSTEMI were older than controls and myocarditis patients. Myocarditis patients had a slightly lower LV EF than controls (55 (49–60)% vs. 64 (62–67)%, *p* < 0.01) and NSTEMI patients (59 (54–64)%, *p* = 0.32). There were no relevant differences in LV end-diastolic volume index (LVEDVi), LV end-systolic volume index (LVESVi), and LV stroke volume index (LVSVi) between the three groups (Table 1).

### Diagnostic performance of the conventional approach

Sensitivity, specificity, and accuracy to detect myocardial infarction were 92% (95%CI: 77–97), 90% (95%CI: 78–96), and 92% (95%CI: 81–97), respectively. Sensitivity, specificity, and accuracy to detect myocarditis were 90% (95%CI: 70–97), 91% (95%CI: 82–96), and 86% (95%CI: 71–94), respectively (Tables 2 and 3).

**Table 2:** Sensitivities and specificities of the different CMR sequences

CMR sequence	NSTEMI		Myocarditis	
	Sensitivity	Specificity	Sensitivity	Specificity
Cine + T2w + LGE	92 (77–97)	90 (78–96)	90 (70–97)	91 (82–96)
Cine	69 (47–84)	71 (50–85)	44 (19–72)	79 (65–89)
T2w	85 (67–94)	79 (61–90)	60 (31–84)	88 (77–94)
LGE	74 (54–88)	86 (71–94)	92 (74–98)	77 (61–87)
T1 maps	85 (67–94)	87 (73–95)	85 (60–96)	87 (75–94)
T2 maps	69 (47–84)	53 (32–72)	48 (21–75)	63 (46–77)

Values are % (95% confidence interval). Cutoff values are listed in parentheses after each parameter

*CMR*, cardiac magnetic resonance; *LGE*, late gadolinium enhancement; *T2w*, T2-weighted cardiac magnetic resonance

**Table 3:** Accuracies of the different CMR sequences and odds ratios of each sequence alone compared to the conventional approach

CMR image	Group	Accuracy	Odds ratio (95%CI)	adjusted *p*-value
Cine + T2w + LGE	Controls	93 (84–97)		
	NSTEMI	92 (81–97)		
	Myocarditis	86 (71–94)		
Cine	Controls	77 (58–89)	0.238 (0.093–0.606)	< 0.001
	NSTEMI	72 (52–86)		
	Myocarditis	60 (38–78)		
T2w	Controls	86 (71–94)	0.435 (0.168–1.131)	0.12
	NSTEMI	83 (66–92)		
	Myocarditis	73 (53–87)		
LGE	Controls	86 (72–94)	0.463 (0.177–1.205)	0.19
	NSTEMI	84 (67–93)		
	Myocarditis	74 (54–87)		
T1	Controls	90 (78–96)	0.638 (0.240–1.700)	1.00
	NSTEMI	88 (74–95)		
	Myocarditis	80 (62–91)		
T2	Controls	67 (46–83)	0.146 (0.057–0.371)	< 0.0001
	NSTEMI	62 (41–79)		
	Myocarditis	47 (28–68)		

Values for accuracies are % (95% confidence interval)

*CI*, confidence interval; *CMR*, cardiac magnetic resonance; *LGE*, late gadolinium enhancement; *NSTEMI*, non-ST elevation myocardial infarction; *T2w*, T2-weighted cardiac magnetic resonance

### Diagnostic performance of cine CMR

Sensitivity, specificity, and accuracy to detect myocardial infarction were 69% (95%CI: 47–84), 71% (95%CI: 50–85), and 72% (95%CI: 52–86), respectively. Sensitivity, specificity, and accuracy to detect myocarditis were 44% (95%CI: 19–72), 79% (95%CI: 65–89), and 60% (95%CI: 38–78), respectively (Tables 2 and 3).

### Diagnostic performance of T2w CMR

Sensitivity, specificity, and accuracy to detect myocardial infarction were 85% (95%CI: 67–94), 79% (95%CI: 61–90), and 83% (95%CI: 66–92), respectively. Sensitivity, specificity, and accuracy to detect myocarditis were 60% (95%CI: 31–84), 88% (95%CI: 77–94), and 73% (95%CI: 53–87), respectively (Tables 2 and 3).

### Diagnostic performance of LGE CMR

Sensitivity, specificity, and accuracy to detect myocardial infarction were 74% (95%CI: 54–88), 86% (95%CI: 71–94), and 84% (95%CI: 67–93), respectively. Sensitivity, specificity, and accuracy to detect myocarditis were 92% (95%CI: 74–98), 77% (95%CI: 61–87), and 74% (95%CI: 54–87), respectively (Tables 2 and 3).

### Diagnostic performance of T1 maps

Sensitivity, specificity, and accuracy to detect myocardial infarction were 85% (95%CI: 67–94), 87% (95%CI: 73–95), and 88% (95%CI: 74–95), respectively. Sensitivity, specificity, and accuracy to detect myocarditis were 85% (95%CI: 60–96), 87% (95%CI: 75–94), and 80% (95%CI: 62–91), respectively (Tables 2 and 3).

### Diagnostic performance of T2 maps

Sensitivity, specificity, and accuracy to detect myocardial infarction were 69% (95%CI: 47–84), 53% (95%CI: 32–72), and 62% (95%CI: 40–79), respectively. Sensitivity, specificity, and accuracy to detect myocarditis were 48% (95%CI: 21–75), 63% (95%CI: 46–77), and 47% (95%CI: 28–68), respectively (Tables 2 and 3).

### Comparison of the different CMR techniques

**Fig. 2:**
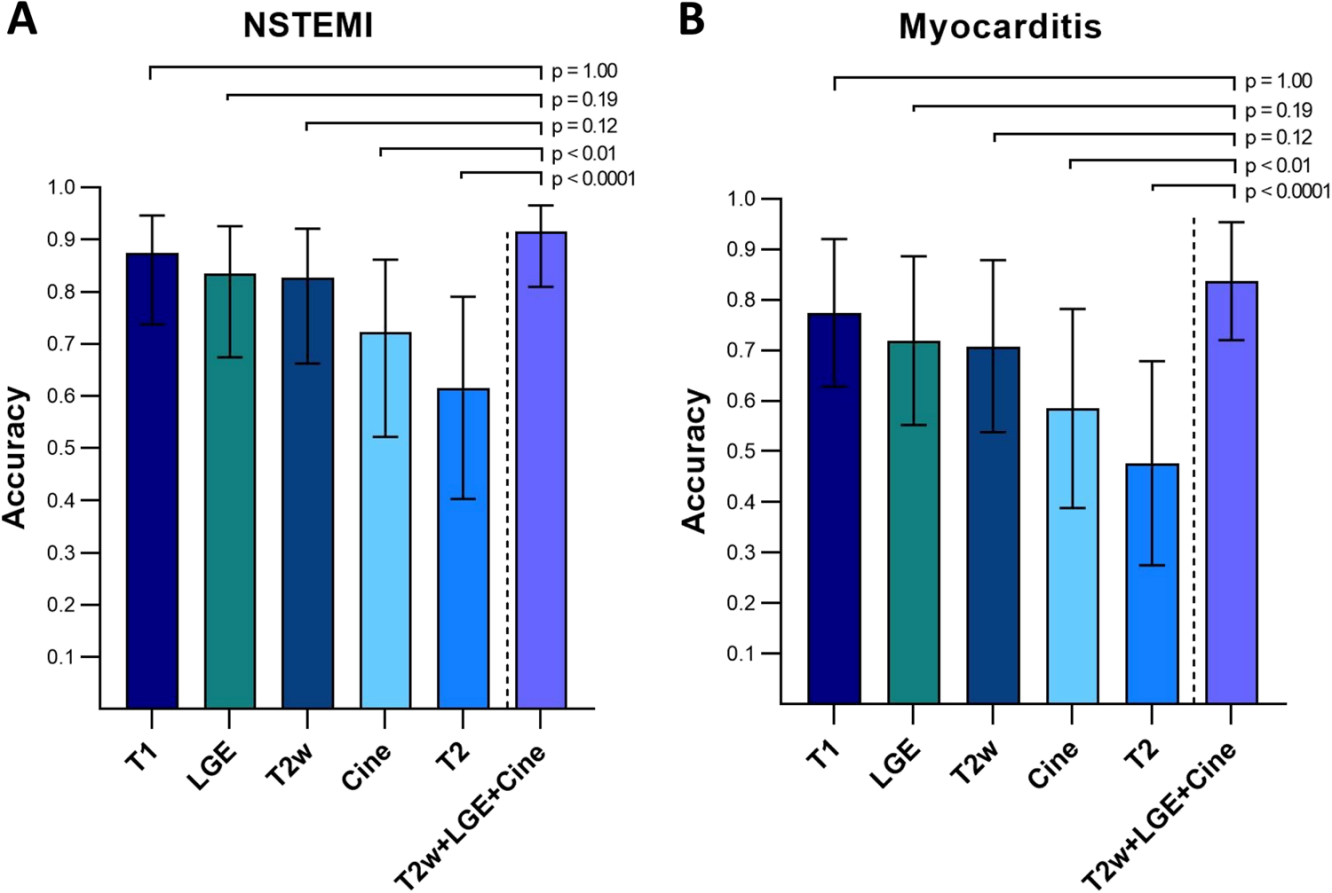
Diagnostic accuracies of the different CMR techniques for NSTEMI (**A**) and myocarditis (**B**). Bars represent overall diagnostic accuracies; error bars indicate 95% confidence intervals. The accuracy for the conventional approach with a combination of cine, T2w and LGE CMR is showed on the right in comparison to all other CMR sequences. *p*-values are given for the likelihood of correctly detecting the target condition compared to the conventional approach

Table 3 and Fig. 2 demonstrate the diagnostic accuracies of the different CMR techniques. The likelihood of correctly detecting the target condition compared to the conventional approach was 0.238 (95%CI: 0.093–0.606, *p* < 0.001) for cine CMR, 0.435 (95%CI: 0.168–1.131, *p* = 0.12) for T2w CMR, 0.463 (95%CI: 0.177–1.205, *p* = 0.19) for LGE imaging, 0.638 (95%CI: 0.240–1.700, *p* = 1.00) for T1 maps, and 0.146 (95%CI: 0.057–0.371, *p* < 0.0001) for T2 maps (Table 3, Fig. 2).

## Discussion

This study analyzed the ability of conventional and parametric CMR techniques to discriminate between NSTEMI, “infarct-like” myocarditis, and controls by visual pattern analysis. The major findings were as follows: First, a visual differentiation of NSTEMI, “infarct-like” myocarditis, and controls by visual pattern analysis can be reliably achieved by the conventional approach with a combination of cine, T2w and LGE imaging; Second, there was no significant difference between a visual pattern assessment of native T1 maps and the conventional approach for differentiating NSTEMI, “infarct-like” myocarditis, and controls. Third, pattern analysis of T2 mapping did not provide sufficient diagnostic accuracy as a stand-alone technique for a visual assessment compared to the combined conventional approach in this context.

### Visual differentiation of NSTEMI and myocarditis by conventional CMR

As indicated above, CMR has an essential role in differentiating ischemic from non-ischemic myocardial disease [2, 4] by displaying edema on T2w images and analyzing the pattern and localization of LGE [2, 4, 18]. Various studies demonstrated that detecting ischemic heart disease by conventional CMR is feasible and reliable [19], especially by an approach of combining conventional sequences as favorized in the current study. Cury et al demonstrated an improvement in the overall accuracy up to 93% and in specificity up to 96% by combining T2w images with a standard protocol of cine, perfusion, and LGE sequences [20, 21]. We found a similar diagnostic accuracy of 92% and specificity of 90% in the current study for detecting myocardial infarction.

Likewise in myocarditis, at least since the establishment of the original LLC in 2009, diagnosis by CMR was based on a combination of different conventional CMR sequences [7]. We found a diagnostic accuracy of 86% for detecting myocarditis by the combined conventional approach which is similar to the diagnostic accuracy of 85% of Abdel-Aty et al who diagnosed myocarditis with any two of three different conventional sequences (EGE, T2w, LGE) and also similar to the diagnostic accuracy of 85% of Luetkens et al who identified acute myocarditis with the established LLC [22, 23]. These accuracies are slightly higher than described in the pooled data analysis by Friedrich et al who found an accuracy of 78% for a combined conventional approach, a finding that was mainly driven by lower accuracies of Gutberlet et al who studied chronic myocarditis and therefore possibly saw a lower diagnostic performance [7, 24]. All in all, these findings highlight the reliable ability of cine, T2w and LGE combined to qualitatively diagnose ischemic and non-ischemic myocardial disease.

### T1 and T2 mapping in myocardial infarction and myocarditis

Nevertheless, there are known limitations of these conventional techniques such as motion artifacts, interfering signal from subendocardial stagnant blood [25, 26], the need for a reference region, and the inability to detect diffuse disease [7, 13]. Several studies demonstrated excellent or even superior diagnostic accuracies using quantitative T1 and T2 measurements in NSTEMI and myocarditis in comparison to LGE and T2w imaging, overcoming hereby many limitations of conventional CMR, even though spatial resolution was better for LGE than for the mapping sequences [13, 25, 27–30]. However, it is important to note that these studies did not address the visual discrimination between ischemic and non-ischemic disease on T1 and T2 maps, which is a crucial task for CMR in patients presenting with non-ST elevation-acute coronary syndrome (NSTE-ACS) [2].

Overcoming the limitations of conventional CMR and replacing it completely, requires a reliable differentiation of ischemic and non-ischemic myocardial injury by means of visual assessment of T1 and T2 maps, similar to LGE imaging [2]. To the best of our knowledge, our study is the first to focus on a sole visual interpretation of T1 and T2 maps in myocardial infarction and “infarct-like” myocarditis.

### Visual assessment of T1 maps

Previous studies like Ferreira et al postulated the potential possibility of quantitative T1 mapping in acute myocarditis to depict typical non-ischemic patterns similar to LGE imaging without the need for a contrast agent [13]. Kali et al characterized chronic myocardial infarction using a threshold-based analysis technique for also T1 maps and found an excellent visual agreement between native T1 mapping and LGE imaging [31]. However, without the threshold-based method T1 maps had high specificity, but modest sensitivity for visual detection of chronic myocardial infarction relative to LGE [31]. The authors explained these findings with the low native T1 contrast between chronic myocardial infarction and remote myocardium resulting in poor diagnostic accuracy [31]. Elevated myocardial T1 values rather represent acute inflammation than chronic injury [14, 32]. This could explain the better diagnostic performance for T1 maps with diagnostic accuracies of 88% to detect myocardial infarction and 80% to detect myocarditis in our study. Moreover, similar to quantitative measurements, spatial resolution appears not to be the decisive factor for visual pattern analyses on T1 and T2 maps. Our findings indicate that a visual assessment of native topographic T1 maps could serve as a clinical tool for differentiation between myocardial infarction, “infarct-like” myocarditis, and controls without the need to obtain quantitative T1 values or conventional CMR sequences.

### Visual assessment of T2 maps

Although Pixel spacing was comparable for the different sequences and a quantitative assessment of T2 has an excellent ability to diagnose both ischemic and non-ischemic disease [14, 15, 25, 27], we found an insufficient performance of a visual assessment of T2 maps compared to conventional CMR with diagnostic accuracies of 62% for myocardial infarction and 47% for myocarditis. A potential explanation for this finding is the relatively small absolute difference in T2 between healthy and diseased myocardium of about 10 to 20 ms, which results in a relatively small difference and poor graduation between injured and remote myocardium on gray-scale or color-coded T2 maps [25, 33, 34]. This inherent limitation of T2 maps most likely impaired the ability to visually appreciate patterns of myocardial injury on the presented color-coded T2 maps compared to T1 maps and conventional imaging.

### Limitations

First, the small size of each cohort limits the performed statistics in our study. Studies with larger sample sizes are needed for further validation. Second, T1 and T2 maps were presented to the observers as three end-diastolic short-axis slices, whereas cine (short-axis cine loops over the entire LV) and LGE (three LV sections in short-axis orientation plus two-, three-, and four-chamber views) covered more sections of the LV anatomy. Therefore, focal myocardial abnormalities might have been missed by the T1 and T2 maps in favor of the conventional approach. However, this approach reflects current routine and recommendations: “Full coverage” of the LV by T1 and T2 maps is clinically not feasible with current sequences and current guidelines recommend restricting T1 and T2 mapping to three slices [10]. Therefore, our data represent a clinically feasible approach to this topic. Third, the current study included only patients with NSTEMI and “infarct-like” myocarditis phenotype. Therefore, the findings of this study are confined to this particular context. Fourth, the interval referring to the timing of CMR in reference to the inciting event for myocarditis was 15 days (IQR 2–27) compared to 8 days (IQR 6–10 days) for NSTEMI. This fact is inevitable in some cases, since patients with myocarditis do have a different symptom burden and course compared to patients with myocardial infarction with subsequent differences in presentation to emergency units.

## Conclusion

The conventional approach with a combination of cine, T2w and LGE CMR provided a reliable visual discrimination between NSTEMI, “infarct-like” myocarditis, and healthy controls. The diagnostic accuracy for a visual pattern analysis of T1 maps was not significantly lower than the conventional approach, whereas the diagnostic accuracy of a visual pattern analysis of T2 maps was not sufficient in this context.

## Abbreviations

CI: Confidence interval
CK: Creatine kinase
CMR: Cardiac magnetic resonance
EF: Ejection fraction
EGE: Early gadolinium enhancement
IQR: Interquartile range
LGE: Late gadolinium enhancement
LLC: Lake Louise criteria
LV: Left ventricular
LVEDVi: Left ventricular end-diastolic volume index
LVESVi: Left ventricular end-systolic volume index
LVSVi: Left ventricular stroke volume index
MOLLI: Modified Look-Locker Inversion Recovery
NSTE-ACS: Non-ST elevation-acute coronary syndrome
NSTEMI: Non-ST elevation myocardial infarction
PSIR: Phase-sensitive inversion recovery
T2w: T2-weighted

## Declarations

### Guarantor

The scientific guarantor of this publication is Enver Tahir.

### Statistics and biometry

One of the authors has significant statistical expertise.

### Study subjects or cohorts overlap

The cohorts included in the current study were previously reported on in the following publications:

Tahir E, Sinn M, Bohnen S et al (2017) Acute versus Chronic Myocardial Infarction: Diagnostic Accuracy of Quantitative Native T1 and T2 Mapping versus Assessment of Edema on Standard T2-weighted Cardiovascular MR Images for Differentiation. Radiology 285 1:83–91.

Bohnen S, Radunski UK, Lund GK et al (2017) Tissue characterization by T1 and T2 mapping cardiovascular magnetic resonance imaging to monitor myocardial inflammation in healing myocarditis. Eur Heart J Cardiovasc Imaging 18 7:744–51.

However, the current study focuses on two distinct subpopulations. These are patients with non-ST elevation myocardial infarction (N = 20) and „infarct-like “ myocarditis (N = 20). Moreover, while the two previous studies addressed quantitative assessment of infarcted and inflamed myocardium using novel T1 and T2 mapping, the current study evaluates different CMR techniques in terms of their qualitative ability to visually differentiate the different patterns of myocardial injury (ischemic vs. non-ischemic).

### Methodology

• retrospective

• observational

• performed at one institution
